# Espiritismo and Santeria: a gateway to child mental health services among Puerto Rican families?

**DOI:** 10.1186/s13034-022-00439-0

**Published:** 2022-01-11

**Authors:** M. Carolina Zerrate, Sara B. VanBronkhorst, Jaimie Klotz, Angel A. Caraballo, Glorisa Canino, Hector R. Bird, Cristiane S. Duarte

**Affiliations:** 1 Division of Child and Adolescent Psychiatry, Columbia University Irving Medical Center, NewYork-Presbyterian, 622 West 168th Street, New York, NY 10032 USA; 2 Division of Child and Adolescent Psychiatry, New York State Psychiatric Institute, Columbia University Irving Medical Center, 1051 Riverside Drive, Unit 43, New York, NY 10032 USA; 3 Division of Child and Adolescent Psychiatry, Columbia University Irving Medical Center, 1051 Riverside Drive, Unit 43, New York, NY 10032 USA; 4 Psychiatrist in Private Practice, 140 W 86th St. 1A Rear, Suite A-4, New York, NY 10025 USA; 5 Behavioral Sciences Research Institute, Medical Sciences Campus, University of Puerto Rico School of Medicine, Office A928 9th Floor, Rio Piedras, PR 00935 USA

**Keywords:** Religiosity and spirituality, Child mental health, Latino or Hispanic, Mental Health Access

## Abstract

**Background:**

Barriers to mental health care access among Latinx children contribute to mental health disparities. It is unclear whether traditional spiritual guides in Latinx communities may function more as gateway providers or in some instances as deterrents to mental health treatment. This study assesses whether family involvement in Espiritismo and/or Santeria, two forefront non-Christian spiritual traditions among Latinx families, is associated with mental health care utilization among Puerto Rican children in two contexts.

**Methods:**

Data are from Waves 1–3 (2000–2004) of the Boricua Youth Study, a population-based longitudinal cohort study of Puerto Rican children from San Juan and Caguas, Puerto Rico (PR), and the South Bronx, New York (SBx), 5 to 17 years of age (N = 2491).

**Results:**

At baseline, 5.02% (n = 58) of the families reported involvement with Espiritismo and/or Santeria in the SBx and 3.64% (n = 52) in PR. Logistic regression models predicting mental health service use found, after adjusting for multiple risk and protective factors, that families involved with Espiritismo and/or Santeria were 2.41 times more likely (*p* = 0.0034) to use mental health services over the course of 3 years than children with no family involvement in these practices in the SBx. The same association was not found in PR.

**Conclusions:**

The findings among PR families in the SBx lend support to the gateway provider model in which spiritual guides open doors to mental health treatment. Forming community connections between mental health providers and traditional spiritual groups may be a culturally considerate, fruitful approach to reducing barriers to mental health treatment among Latinx families.

## Background

The Hispanic, or Latinx, population is the largest minority group in the United States, and it is estimated that by the year 2060 over 30% of children under the age of 18 will be Hispanic [1]. Almost one-third of Latinx children are living in families with incomes below the poverty line [2]. Social disparities, including health care access for Latinx children, are a growing concern. Latinx children are the largest group among racial/ethnic marginalized minority and non-minority children who are eligible, but not enrolled to receive health care benefits [3]. Mental health care utilization, specifically, is lower among Latinx children in the United States compared to White children [4, 5]. A report from a nationally representative sample found that service utilization among Latinx children with mental disorders is 50% lower than non-Hispanic White children [6]. Among Latinx subgroups in the United States, Puerto Rican adults have been identified as having higher rates of mental health service utilization, but also higher rates of mental disorders relative to other Latinx subgroups [7–9]. Puerto Ricans have also faced increased stressors in recent years which may exacerbate mental health problems. The hurricane season in 2017 led to a sustained economic depression in Puerto Rico, with increased migration to the mainland United States; and the COVID-19 pandemic has led to ongoing devastation in Latinx communities. It is, therefore, important to understand successful pathways to mental health treatment among Puerto Ricans and reduce barriers to accessing treatment.

Barriers to accessing mental health treatment for Latinx children include stigma related to mental health treatment and lack of confidence in treatment options, in addition to larger systemic factors leading to reduced availability of resources [10]. To understand and address barriers to mental health treatment in groups that have been marginalized, it is important to consider strategies that incorporate cultural humility and competence [11]. To begin, we suggest examining pathways of referral to mental health services for Latinx children. The ability of adults to recognize emotional or behavioral problems in children, together with knowledge of available mental health services, contribute to service utilization among children and adolescents [12–14]. In addition to parents and health care providers, other adults in the community may serve either as obstacles, by deterring the use of mental health services, or as liaisons, by encouraging service seeking or by providing mental health service referrals. Stiffman et al. proposed a model to frame treatment seeking behavior, where those who liaise for service access are identified as ‘gateway providers’ [15]. In the Gateway Provider Model, adults who are able to identify signs or symptoms of mental illnesses open doors for mental health treatment by prompting or initiating the referral process. Among groups that have been marginalized, mental health stigma, financial strains, and language barriers around mental health care are some of the obstacles to engagement in mental health treatment [16, 17]. In these communities, spiritual leaders or spiritual guides may absorb some of the demand for mental health care. Barriers and stigma associated mental health treatment may be lower in the personal, long-term, relationships established with spiritual leaders. Several reports focusing on populations that have been marginalized around the world have highlighted the role of spiritual leaders as gateway providers [13, 18]. Furthermore, collaborations between biomedical providers and spiritual leaders have demonstrated positive results in mental health treatment [19].

Among Latinx groups in the United States, spiritual leaders, when well informed about mental illness and available treatments, can play the role of gateway providers [13, 18]. Spiritual beliefs are a central part of the lives of many Latinx families in the United States [20]. Thus, spiritual leaders can potentially have a meaningful and influential role in decreasing the mental health treatment gap for Latinx children.

Santeria and Espiritismo are the two foremost non-Christian spiritual traditions that individuals from Puerto Rico, Dominican Republic, Cuba and their descendants practice in the United States. Espiritismo is defined as a spiritual belief-system based upon the reconciliation of nineteenth century French spiritualism and, for Puerto Ricans, the Taino tribes’ healing practices. Unlike a ‘formal’ religion, spiritual belief-systems can be religious, ideological, philosophical, or a combination of these. Espiritismo is based on the idea that the visible and invisible world are inhabited by spirits, and temporarily encased in a human body in the material world. Santeria is a religion which combines ancient West-African religious practices of the inhabitants of the Yoruba tribe with Catholicism. Espiritismo and Santeria are related to supernatural beliefs, such as the ability to communicate with spirits or ancestors to receive guidance [21, 22]. Due to a tradition of secrecy behind these practices, individuals may not disclose involvement in either of these traditions. While the prevalence of Espiritismo practitioners is not known, the American Religious Identification Survey of 2001 estimated that there were approximately 22,000 practitioners of Santeria in the United States [23].

Both Santeria and Espiritismo can be a source not only of spiritual support but also serve as an informal source of support for individuals with mental disorders or enduring emotional or psychological distress [24, 25]. Within the Espiritismo and/or Santeria traditions, spiritual seers, or guides, are called Espiritistas or Santeros and are thought to have the ability to communicate between the spirits of the living and the spirits of the dead [26]. Espiritistas and Santeros can provide remedies or perform rituals to treat physical and emotional problems. An Espiritista or Santero may be perceived as having less cultural stigma for Puerto Rican families compared to consulting a psychologist or psychiatrist [27].

The relationship between spiritual beliefs and utilization of mental health services depends on numerous factors. Compatibility of belief systems with the biomedical understanding of mental illness and mental health treatment, characteristics of the individuals, cultural, and contextual factors all play a role in the influence of spiritual beliefs on utilization of mental health treatment. The relationship between engagement in Espiritismo and/or Santeria and utilization of mental health care is unclear. There are varying reports of the compatibility of the belief system with the biomedical understanding of disease etiology and treatment. For example, one study found that in Espiritismo traditions, while patients are urged to consult physicians and take prescribed medications to relieve physical symptoms, disturbances of feelings or interpersonal relationships are treated by the Espiritistas themselves [21]. On the other hand, a 1990 study by Hohmann et al. found that Puerto Ricans who visited Espiritistas were more likely to seek help for emotional problems from mental health professionals compared to those that had never consulted an Espiritista [28]. There are also reports of clinicians who have collaborated with Santeros, on behalf of mutual patients. Santeros have advised patients to take medications, get lab tests, and keep follow-up appointments. They have also utilized assessments created by mainstream health professionals to make therapeutic goals more meaningful and treatment techniques more compatible with the Santeria belief system [29]. Additionally, understanding individual characteristics of those who are involved in Espiritismo and/or Santeria may help understand any potential differences in mental health care utilization. For example, in the study mentioned above by Hohmann, Richeport [28], adult Puerto Ricans (residing on the island) who visited Espiritistas were not only more likely to have symptoms of depression and seek help for emotional problems from mental health professionals, they were also more likely to work outside the home and to have low family income [28]. Furthermore, it is unclear how characteristics of Puerto Ricans engaging in Espiritismo and/or Santeria living as a group that has been marginalized (in the South Bronx [SBx], for example) would compare to those engaging in Espiritismo and/or Santeria who reside in Puerto Rico.

This paper aims to address the different findings in the literature by examining the association between involvement of a relative with Espiritismo and/or Santeria and child mental health service use in Puerto Rican families living in two different contexts: San Juan, PR (PR) and SBx, New York City. The main question that the present study is designed to answer is if Puerto Rican families involved in Espiritismo and/or Santeria are more likely to utilize mental health services, revealing a pattern compatible with spiritual guides acting as gateway providers in the community. The inclusion of two sites in this study provides a unique opportunity to examine how cultural context may influence the relationship between Espiritismo and/or Santeria involvement and child mental health service use. In addition, we aim to identify characteristics among these families involved in Espiritismo and/or Santeria that may impact the use of child mental health services.

## Methods

This paper reports on data from the Boricua Youth Study, a longitudinal study of Puerto Rican children in two contexts: the SBx and the Standard Metropolitan Areas in San Juan and Caguas, PR (total sample size N = 2491).

### Sample

Puerto Rican children aged 5–13 years at Wave 1 residing in the SBx (n = 1138) and in PR (n = 1351) were assessed annually over three waves of data collection. As described by Bird, Canino [30], each sample is a multistage probability sample of households of the target population, and each can be weighted to represent the populations of Puerto Rican children in the SBx and those in PR. A household met inclusion criteria if: (1) there was at least one child residing in the household aged 5 through 13 years; and (2) at least one of the child’s parents or primary caretakers self-identified as being of Puerto Rican background. All eligible children were selected to participate up to a maximum of three children per household. In households with more than three eligible children, three were selected at random. Children with a diagnosis of mental retardation or a developmental disability and children not residing in the household for at least the previous 9 months were excluded. Three assessments, 1 year apart each, were conducted from 2000 to 2004 (Waves 1 to 3).

### Procedure

Families were enrolled in the study after parents provided consent for all children and children over age 7 years provided assent. Consent forms and procedures were approved by the New York State Psychiatric Institute I.R.B. and by the I.R.B. at the University of Puerto Rico Medical School. Participants and their primary caretakers were interviewed using a wide array of risk factor measures. Interviews were conducted in the participant’s home using laptop computers. Caretakers and children were interviewed separately, to the extent possible. The items of interest for the present analysis were reported by the primary caretakers only. Interviews were administered in either Spanish or English. Participants could respond in either language and switching between languages was permitted. For quality control, interviews were audiotaped with the consent of parent and child and were later spot-checked for quality control. Additional information about the methodology and initial findings has been provided by Bird, Canino [30].

### Measures

#### Child mental health service use

Use of inpatient, outpatient, day treatment, special education, and school counseling for mental health services due to emotional, behavioral, alcohol, or drug problems during the previous year was ascertained [31]. Caretaker reports documented at Waves 1, 2 and 3 were utilized in this analysis.

#### Involvement in Espiritismo or Santeria by a family member

Defined based on caretaker’s answers to two questions at Wave 1: “Is anyone in the immediate family involved in Espiritismo?” and “Is anyone in the immediate family involved in Santeria?”.

##### Correlates

Socio-demographic information: Child gender, age, maternal education, and parental nativity (if both parents were born in PR or other Latin country, or if one of them was born in the US or other non-Latin country) were documented at Wave 1.

Psychiatric diagnosis and impairment: Presence of any psychiatric diagnosis was determined using the Diagnostic Interview Schedule for Children (DISC-IV) [32, 33]. The DISC was not administered to children under 10 years because the reliability of younger child informants is questionable. To be consistent, cases of all ages were defined using only data obtained from the adult informant on the DISC-IV, even though children who were 10 years or older were also administered the DISC-IV. The following diagnoses were ascertained: conduct disorder and oppositional defiant disorder, attention deficit hyperactivity disorder, major depression, dysthymia, anxiety disorders (separation anxiety, panic and generalized anxiety disorders, social phobia and post-traumatic stress disorder) and substance use disorder. In addition to meeting the impairment criteria for diagnosis as per DSM-IV-TR, functional impairment was also ascertained with the Children’s Global Assessment Scale (C-GAS). The C-GAS provides a global measure of level of functioning on a scale from 1 to 100, independent of diagnostic criteria [34].

Parental psychopathology: Parental psychopathology was assessed by means of the Family History Screen for Epidemiologic Studies (FHE) [35, 36], which screens for lifetime history of psychiatric illness in primary caregivers. The FHE was utilized to screen for a selected group of conditions including depression, substance use, antisocial behavior and suicide attempts. Parental psychopathology was defined as possibly present if a paternal or maternal figure screened positive for any condition included in the FHE. The psychometric studies for the FHE [36] revealed satisfactory test–retest reliability for self-report on the psychiatric illnesses included (kappa > 0.56, for self-reports 15 months apart) and sensitivity ranging from 56.0 to 86.8 and specificity ranging from 65.0 to 93.5 for best estimate diagnosis (8 clinicians).

Parental social support: Social support was assessed through caretaker’s answers to six different questions (Cronbach’s alpha = 0.71) assessing support from family members, neighbors, and friends.

Physical health services: Use of health services for physical health issues was assessed through caretakers’ answers on three different questions about use of outpatient medical services in the past 12 months.

### Statistical methods

Weighted percentages, means, and standard errors of baseline demographic, mental health, social support, and service use variables were calculated for the study sample, stratified by site and involvement in Espiritismo and/or Santeria. Chi-squared estimates (for categorical variables) and t-tests (for continuous variables) were used to look for associations between sample characteristics and family involvement in Espiritismo and/or Santeria within PR and the SBx separately. Next, we estimated the prevalence rates of child mental health service use and Espiritismo and/or Santeria involvement at all three waves, using Chi-square statistics to determine possible associations within each site at each wave.

To investigate the longitudinal relationship between involvement in Espiritismo and/or Santeria and child mental health service use, we used a series of nine logistic regression models. After starting with the unadjusted model (Model 1), we added demographic covariates (gender, age, and maternal education) (Model 2). Models 3 through 8 each added a single variable to Model 2: (3) parental nativity, (4) any child psychiatric diagnosis, (5) child impaired functioning, (6) parental probable psychopathology, (7) parental social support, and (8) past year physical health service use. A final model (Model 9) adjusted for all variables of interest.

All analyses incorporated sampling weights to account for study design. We used generalized estimating equations (GEE) for our logistic regression models to take into account dependencies among repeated measures across the three waves [37]. Missing data were handled using listwise deletion for variables used in each individual model. Statistical significance was assessed at an alpha level of 0.05. All analyses were conducted using SAS 9.4 (SAS Institute Inc., Cary, North Carolina).

## Results

### Descriptive results

The prevalence of family involvement in Espiritismo and/or Santeria was not statistically different between sites and was similar across the three waves. A total of 5.02% (n = 58) of participants in the SBx were involved in Espiritismo and/or Santeria compared to 3.64% (n = 52) in PR (*p* = 0.15) at Wave 1. Factors associated with Espiritismo and/or Santeria are presented by site in Table 1. In the SBx, families involved with Espiritismo and/or Santeria more frequently had a parent with probable psychopathology (*p* < 0.001) and had a lower level of parental social support (*p* = 0.024). In PR, families involved with Espiritismo and/or Santeria were similarly high on probable parental psychopathology (*p* = 0.001). Unlike in the SBx, in PR there was no difference in parental social support between groups (*p* = 0.351). Additionally, in PR, families involved in Espiritismo and/or Santeria more frequently had parents who were both Puerto Rican (*p* = 0.047), had older children (*p* = 0.024), and had a child with a psychiatric diagnosis (*p* = 0.003). There were no statistically significant differences in either site in relation to maternal education level, the degree of child impairment as measured by the C-GAS, or use of health service in the past 12 months.

**Table 1:** Selected factors and family member involvement with Espiritismo and/or Santeria at baseline by site

Sample characteristics	Family member involvement with Espiritismo and/or Santeria					
	South Bronx			Puerto Rico		
	No (n = 1077)	Yes (n = 58)	p value	No (n = 1298)	Yes (n = 52)	p value
%		5.02			3.64	0.150^1^
Gender (%)						
Female	49.2	44.7	0.562^a^	48.6	57.6	0.297^a^
Age [mean (SE)]	9.25 (0.08)	8.9 (0.34)	0.315^b^	9.14 (0.08)	10.01 (0.33)	**0.024**^b^
Maternal education (%)						
< High school	46.8	39.4		24.7	17.5	
High school	43.1	46.8		43.2	43.6	
College	10.2	13.8	0.519^a^	32.1	38.9	0.543^a^
Parental nativity (%)						
Both Puerto Rican	30.2	18.5		79.5	91.5	
One American	69.8	81.5	0.056^a^	20.5	8.5	**0.047**^**a**^
Any child psychiatric diagnosis (%)						
Yes	8.7	6.3	0.526^a^	9.1	24.7	**0.003**^**a**^
Child impaired functioning (C-GAS) (%)						
< 69	15.1	18.9	0.434^a^	15.7	19.8	0.481^a^
Parental probable psychopathology (%)						
Yes	32.5	63.1	**< 0.001**^**a**^	38.2	67.1	**0.001**^**a**^
Parental social support [mean (SE)]	1.07 (0.02)	0.92 (0.07)	**0.024**^**b**^	1.80 (0.02)	1.74 (0.04)	0.351^b^
Health service use past 12 m [mean (SE)]	3.32 (0.12)	4.11 (0.61)	0.212^b^	3.79 (0.13)	4.20 (0.59)	0.554^b^

Boldface indicates *p* < 0.05

*C-GAS* Children’s Global Assessment Scale

^a^Chi-square test

^b^*t*-test

In the SBx, a positive association between family member involvement with Espiritismo and/or Santeria and the use of child mental health service was found. At Wave 1, 18.4% of 1077 families not involved with Espiritismo and/or Santeria reported use of child mental health services, while 35.5% of 58 families involved with Espiritismo and/or Santeria reported use of child mental health services (*p* = 0.006). The same was not true in PR, as shown in Table 2; 12.6% of 1298 families not involved with Espiritismo and/or Santeria reported use of child mental health services, vs. 10.4% of 52 families who were involved with Espiritismo and/or Santeria (*p* = 0.718). At two subsequent follow-ups similar patterns persisted within each site; however, at Wave 3 the associations in the SBx were no longer statistically significant (*p* = 0.088).

**Table 2:** Family member involvement with Espiritismo and/or Santeria among participants with child mental health service use over three study waves by site

Child mental health service use	Family member involvement with Espiritismo and/or Santeria									
	South Bronx					Puerto Rico				
	No		Yes		p	No		Yes		p
	n	%	n	%		n	%	n	%	
Wave 1	204	18.4	20	35.5	**0.006**	171	12.6	7	10.4	0.718
Wave 2	219	21.8	16	36.0	**0.036**	175	14.2	6	15.8	0.811
Wave 3	202	21.0	13	33.0	0.088	159	13.6	6	14.6	0.869

Boldface indicates p < 0.05

### Multivariate models of longitudinal data

We considered the impact of multiple factors across the three waves on the association between service use and family involvement with Espiritismo and/or Santeria in each site. As demonstrated in Table 3, family involvement with Espiritismo and/or Santeria was positively associated with child mental health service use in the SBx in all of the models analyzed. The model testing the effect of a child psychiatric diagnosis yielded the highest odds ratio (aOR = 2.28, 95% CI [1.30, 4.01]), of all the models analyzed. The final model (Model 9), demonstrated that after controlling for all included sociodemographic and predictive factors, children in the SBx with a family member involved with Espiritismo and/or Santeria had a higher odds (aOR = 2.41; *p* = 0.0034) of using mental health services over the course of 3 years compared to children with no family involvement in these practices. Family involvement with Espiritismo and/or Santeria was not statistically associated with child mental health service use in PR in any of the models.

**Table 3:** Family member involvement with Espiritismo and/or Santeria at wave 1 and child mental health service use over time

Estimates for Espiritismo and/or Santeria (Wave 1)	Child mental health service use over three study waves					
	South Bronx			Puerto Rico		
	Regression coefficient^a^	aOR (95% CI)	p value	Regression coefficient^a^	aOR (95% CI)	p value
Model 1: Family member involvement with Espiritismo and/or Santeria	0.72	2.06 (1.24, 3.43)	**0.0054**	− 0.04	0.96 (0.50, 1.85)	0.7097
Model 2: Model 1 + gender/age/maternal education	0.71	2.04 (1.17, 3.55)	**0.0117**	0.12	1.13 (0.60, 2.11)	0.7097
Model 3: Model 2 + parental nativity	0.73	2.08 (1.19, 3.63)	**0.0101**	0.06	1.06 (0.56, 1.99)	0.8572
Model 4: Model 2 + any child psychiatric diagnosis	0.82	2.28 (1.30, 4.01)	**0.0042**	− 0.21	0.81 (0.46, 1.40)	0.4479
Model 5: Model 2 + child impaired functioning	0.73	2.07 (1.17, 3.66)	**0.0126**	0.04	1.04 (0.60, 1.82)	0.8808
Model 6: Model 2 + parental probable psychopathology	0.63	1.88 (1.08, 3.26)	**0.0251**	− 0.08	0.93 (0.50, 1.73)	0.8105
Model 7: Model 2 + parental social support	0.67	1.96 (1.12, 3.41)	**0.0183**	0.12	1.13 (0.60, 2.11)	0.7020
Model 8: Model 2 + health service use past year	0.67	1.96 (1.14, 3.36)	**0.0145**	0.08	1.08 (0.58, 2.01)	0.8092
Model 9: All	0.88	2.41 (1.34, 4.35)	**0.0034**	− 0.38	0.69 (0.39, 1.22)	0.2016

Boldface indicates p < 0.05

*OR* odds ratio

^a^Derived from generalized estimating equations (GEE)

## Discussion

This study aimed to understand how Espiritismo and/or Santeria involvement among Puerto Rican families may be related to child mental health care utilization and how this relationship may be influenced by cultural context. We found that in the SBx, but not in PR, children whose families were involved with Espiritismo and/or Santeria were more likely to use mental health services compared to families without this involvement. This finding was not explained by other study variables, including child psychiatric disorders, parental social support, or health service use. Our results are consistent with the notion that spiritual guides in populations that have been marginalized can function as gateway providers for mental health care treatment.

The prevalence of family involvement in Espiritismo and/or Santeria was similar in the SBx (5.02%) and in PR (3.64%), indicating that these traditional spiritual practices have been maintained in a new cultural context. Involvement with Espiritismo and/or Santeria was associated with probable parental psychopathology in both sites and with child psychiatric diagnosis in PR. This likely reflects the use of Espiritismo and/or Santeria to address mental health problems and suggests that Espiritistas and Santeros encounter many people seeking mental health treatment. Therefore, offering referral information and initiating conversations with these spiritual leaders could prove beneficial. Finally, in the SBx, but not in PR, lower levels of social support were reported by families involved with Espiritismo and/or Santeria compared to those without this involvement. Puerto Ricans in the SBx often have disrupted social support systems due to the back-and-forth patterns of migration and the separation from extended family [38]. For these families, Espiritismo and/or Santeria involvement may provide a form of social support consistent with their cultural heritage.

In the SBx, we found a positive and statistically significant association between having a family member involved with Espiritismo and/or Santeria at the time of the first and second interviews (Waves 1 and 2) and child mental health service use over 3 years. The association in Wave 3 was only marginally significant (*p* = 0.088); however, the direction of the association remained consistent and the lack of significance was likely due to attrition of participants across waves. Our findings are compatible with the notion that spiritual guides within the Espiritismo and/or Santeria tradition in the SBx may function as gateway providers to mental health treatment. While similar studies among non-clinical samples are lacking, our findings are consistent with studies among clinical samples in the 1970s of PR families in New York which found that it was very common for families seeking mental health treatment to be involved in Espiritistas practices [26].

We found that context was important in predicting the relationship between involvement in Espiritismo and/or Santeria and child mental health treatment, as the association was significant in the SBx, but not in PR. These results echo findings from prior studies that have suggested that the role of religious guides as gateway providers is particular to groups which have formerly been described as “minority groups” [13, 18]. It may be possible that cultural norms for mental health treatment in PR have evolved in way that they have not evolved in the SBx. That is, while in the past it may have been common for families to seek care from both mental health clinics as well as from Espiritistas and/or Santeros [26], in PR these treatment modalities may have evolved to become more siloed and less mutually compatible treatment modalities. In contrast, in the SBx, the tradition of seeking care from both traditional spiritual healers and mental health clinics may have remained the same, reflecting a preservation of this approach to mental health problems. As described in a detailed history of Santeria by Brandon [39], the evolution of spiritists beliefs and practices are influenced by cultural context.

After adjusting for relevant factors such as the prevalence of a child psychiatric disorder, social support, and the use of medical services for non-mental health problems, those involved with Santeria/Espiritismo in the SBx had a 2.41 higher odds of using mental health services compared to those not involved with Espiritismo and/or Santeria. Our results align with findings by Hohmann, Richeport [28] who described that, among Puerto Rican families residing in the island, adults involved with Espiritistas were more likely to seek help for emotional problems from mental health professionals compared to the those who were not involved with Espiritistas.

The role of religious leaders and traditional healers in promoting access to mental health service and treatment among children and adolescents has also been documented in different communities around the world. A study among adolescent Somali refugees in the United States found that religious leaders functioned as gateway providers [18]. Similarly, a cross-sectional study of children and adolescents in Egypt showed that a substantial group of families of patients with mental health issues were referred by traditional healers to mental health services [40]. In a qualitative study of Canadian youth who attempted suicide, religious community members were also identified as a bridge for the pursuit of mental health services [41].

There is growing interest and information about the role of spiritual guides and traditional healers not only as gateway providers, but also as collaborators in mental health treatment. Collaborating with traditional spiritual guides who see benefits in using a biomedical approach to address emotional and behavioral disorders could help increase access to mental health care for marginalized groups. This collaborative approach could provide a more integrated and culturally centered treatment. Establishing relationships with local spiritual guides could begin by expressing interest in learning about their cultural and spiritual understanding of emotional and behavioral disorders and sharing the biomedical perspective. Within these relationships, identifying convergence points to identify youth who would benefit from mental health treatment would not only increase access to care but help biomedical providers to incorporate cultural strengths in to their treatment plans, thus creating a more genuine patient-centered experience [42]. This collaborative approach between biomedical treatment and spiritual or traditional healers for mental illness has been explored by other groups, particularly in settings where the gaps in mental health treatment are devastating, including in low and middle income countries (LMIC) [43]. Uwakwe and Otakpor [44] proposed incorporating traditional healers not only as gateway providers to scale up referrals for mental health treatment, but as a support to biomedical treatments in countries where disparities are the greatest. These initiatives are not circumscribed to LMIC. Hills et al. [19] described how, with thoughtful engagement, medical health practitioners and practitioners from African healing traditions in East London established close communication and exchange about the treatment of African Caribbean immigrants living in London. Luchetti et al. [45] described collaborations with spiritual leaders in Brazil in the form of psychiatric hospitals identified as Spiritist Psychiatric Hospitals where patients are offered spiritist treatments complimentary to medical treatment for mental illness while they are hospitalized. These collaborations fully embrace the practice of cultural humility and competence which is essential to develop a culturally-centered relationship with youth and families that facilitates understanding of different belief systems. Clinicians working with Latinx communities in the United States are encouraged to engage in this practice, and when available reach out to spiritual guides and other community members that could serve as gateway providers, with the hope of increasing child mental health service use and supporting the treatment for those who need it.

Our study is limited by the lack of knowledge about how our findings may generalize to other Latinx ethnic subgroups besides Puerto Rican children and generalize to clinical populations. Some participants may not have disclosed involvement with Espiritismo and/or Santeria due to potential perceived stigma. However, we do not suspect that the decision to report this would have been influenced by the study outcome variable and therefore it is not likely to have influenced our results. The modest sample size of families involved with Espiritismo and/or Santeria limited our ability to do subgroup analyses. For example, we were not able to examine potential factors influencing child mental health treatment among those involved with Espiritismo and/or Santeria such as the extent of involvement or the presence of an affiliation with a Christian religion. As in other epidemiological studies, despite the longitudinal design and adjustment for potential confounders it is not possible to establish that the involvement with Espiritismo and/or Santeria leads to mental health service use. Other factors not examined in our study, such as parents seeking out Espiritismo and/or Santeria involvement as a form of treatment for subclinical mental health problems could be influencing the results. Finally, relatively little is known regarding how contemporary traditional spiritual practices align with mental health treatment and many questions remain. Future studies could benefit from utilizing a qualitative or mixed-method approach to investigate questions such as why families are involved with Espiritismo and/or Santeria, if these spiritual practices may vary across sites, and how these characteristics may influence the decision of families to seek mental health treatment for their children.

## Conclusion

There is a significant disparity in access and service utilization for treatment of mental health disorders among Latinx children compared to non-Hispanic White children in the United States. Our results show that Puerto Rican families in the SBx who are involved in either Santeria or Espiritismo have a higher odds of using child mental health services compared to those who are not involved in these practices. Spiritual guides can play the role of gateway providers, representing the first step towards accessing mental health care services, and can work in collaboration with biomedical providers to promote patient-centered treatment. As such, Santeros and Espiritistas can facilitate evaluation and add to the biomedical treatment of children with mental health issues. Identifying and reaching out to gateway providers within populations that have been marginalized may provide a solid foundation for developing a culturally informed and financially viable pathway to increase access to mental health services. This approach aims to diminish the treatment gap and improve the lives of Latinx children and adolescents in the United States.

## Data Availability

The Boricua Youth Study data used in this report are available upon on request.

## Abbreviations

SBx: South Bronx
PR: Puerto Rico
DISC-IV: Diagnostic Interview Schedule for Children
FHE: Family History Screen for Epidemiologic Studies

## References

[1] Colby SL, Ortman JM. Projections of the size and composition of the US population: 2014 to 2060. Population estimates and projections. Current population reports. P25–1143. US Census Bureau. 2015.

[2] Nolan L, Garfinkel I, Kaushal N, Nam J, Waldfogel J, Wimer C (2016). Trends in child poverty by race/ethnicity: new evidence using an anchored historical supplemental poverty measure. J Appl Res Child Inf Policy Child Risk.

[3] Scott G, Ni H (2004). Access to health care among Hispanic/Latino children: United States, 1998–2001. Adv Data.

[4] Lopez C, Bergren MD, Painter SG (2008). Latino disparities in child mental health services. J Child Adolesc Psychiatr Nurs.

[5] Cook BL, Barry CL, Busch SH (2013). Racial/ethnic disparity trends in children's mental health care access and expenditures from 2002 to 2007. Health Serv Res.

[6] Merikangas KR, He J-P, Brody D, Fisher PW, Bourdon K, Koretz DS (2010). Prevalence and treatment of mental disorders among US children in the 2001–2004 NHANES. Pediatrics.

[7] Keyes KM, Martins SS, Hatzenbuehler ML, Blanco C, Bates LM, Hasin DS (2012). Mental health service utilization for psychiatric disorders among Latinos living in the United States: the role of ethnic subgroup, ethnic identity, and language/social preferences. Soc Psychiatry Psychiatr Epidemiol.

[8] Alegría M, Mulvaney-Day N, Woo M, Torres M, Gao S, Oddo V (2007). Correlates of past-year mental health service use among Latinos: results from the National Latino and Asian American Study. Am J Public Health.

[9] Alegría M, Canino G, Shrout PE, Woo M, Duan N, Vila D (2008). Prevalence of mental illness in immigrant and non-immigrant U.S. Latino groups. Am J Psychiatry.

[10] Pumariega AJ, Rogers K, Rothe E (2005). Culturally competent systems of care for children’s mental health: advances and challenges. Community Ment Health J.

[11] Pumariega AJ, Rothe E, Mian A, Carlisle L, Toppelberg C, Harris T (2013). Practice parameter for cultural competence in child and adolescent psychiatric practice. J Am Acad Child Adolesc Psychiatry.

[12] Bird HR, Shrout PE, Duarte CS, Shen S, Bauermeister JJ, Canino G (2008). Longitudinal mental health service and medication use for ADHD among Puerto Rican youth in two contexts. J Am Acad Child Adolesc Psychiatry.

[13] Stiffman AR, Freedenthal S, Dore P, Ostmann E, Osborne V, Silmere H (2006). The role of providers in mental health services offered to American-Indian youths. Psychiatr Serv.

[14] Teagle SE (2002). Parental problem recognition and child mental health service use. Ment Health Serv Res.

[15] Stiffman AR, Pescosolido B, Cabassa LJ (2004). Building a model to understand youth service access: the gateway provider model. Ment Health Serv Res.

[16] Caplan S (2019). Intersection of cultural and religious beliefs about mental health: Latinos in the faith-based setting. Hispanic Health Care Int.

[17] Moreno O, Nelson T, Cardemil E (2017). Religiosity and attitudes towards professional mental health services: analysing religious coping as a mediator among Mexican origin Latinas/os in the southwest United States. Ment Health Relig Cult.

[18] Ellis BH, Lincoln AK, Charney ME, Ford-Paz R, Benson M, Strunin L (2010). Mental health service utilization of Somali adolescents: religion, community, and school as gateways to healing. Transcult Psychiatry.

[19] Hills D, Aram E, Hinds D, Warrington C, Brissett L, Stock L (2013). Traditional Healers research project. Final report of the Tavistock Institute of Human Relations.

[20] Pew Research Center (2007). Religious practices and beliefs.

[21] Garrison V (1977). Part three: Doctor, espiritista or psychiatrist?: Health-seeking behavior in a Puerto Rican neighborhood of New York city. Med Anthropol.

[22] Harwood A (1977). Rx: Spiritist as needed: a study of a Puerto Rican community mental health resource.

[23] Kosmin BA, Mayer E, Keysar A. American religious identification survey 2001. 2001.

[24] Rivera ET (2005). The flywheel of the Puerto Rican spiritual traditions. R Interam Psicol.

[25] Rosario AM, De La Rosa M (2014). Santería as informal mental health support among US Latinos with cancer. J Relig Spirit Soc Work Soc Thought.

[26] Bird HR, Canino I (1981). The sociopsychiatry of espiritismo: findings of a study in psychiatric populations of Puerto Rican and other Hispanic children. J Am Acad Child Psychiatry.

[27] Salgado RM. The role of the Puerto Rican spiritist in helping Puerto Ricans with problems of family relations. ProQuest Information & Learning; 1974.

[28] Hohmann AA, Richeport M, Marriott BM, Canino GJ, Rubio-Stipec M, Bird H (1990). Spiritism in Puerto Rico. Results of an island-wide community study. Br J Psychiatry.

[29] Sandoval MC (1979). Santeria as a mental health care system: an historical overview. Soc Sci Med Part B Med Anthropol.

[30] Bird HR, Canino GJ, Davies M, Duarte CS, Febo V, Ramirez R (2006). A study of disruptive behavior disorders in Puerto Rican youth: I. Background, design, and survey methods. J Am Acad Child Adolesc Psychiatry.

[31] Leaf PJ, Alegria M, Cohen P, Goodman SH, Horwitz SM, Hoven CW (1996). Mental health service use in the community and schools: results from the four-community MECA study. J Am Acad Child Adolesc Psychiatry.

[32] Bravo M, Ribera J, Rubio-Stipec M, Canino G, Shrout P, Ramírez R (2001). Test-retest reliability of the Spanish version of the Diagnostic Interview Schedule for Children (DISC-IV). J Abnorm Child Psychol.

[33] Shaffer D, Fisher P, Lucas CP, Dulcan MK, Schwab-Stone ME (2000). NIMH Diagnostic Interview Schedule for Children Version IV (NIMH DISC-IV): description, differences from previous versions, and reliability of some common diagnoses. J Am Acad Child Adolesc Psychiatry.

[34] Shaffer D, Gould MS, Brasic J, Ambrosini P, Fisher P, Bird H (1983). A children's global assessment scale (CGAS). Arch Gen Psychiatry.

[35] Lish JD, Weissman MM, Adams PB, Hoven CW, Bird H (1995). Family psychiatric screening instrument for epidemiologic studies: pilot testing and validation. Psychiatry Res.

[36] Weissman MM, Wickramaratne P, Adams P, Wolk S, Verdeli H, Olfson M (2000). Brief screening for family psychiatric history: the family history screen. Arch Gen Psychiatry.

[37] Diggle P (2002). Analysis of longitudinal data.

[38] Garcia-Preto N. Puerto Rican families. In: Ethnicity and family therapy. Vol. 3. 1982.

[39] Brandon G (1997). Santeria from Africa to the New World: the dead sell memories.

[40] Hussein H, Shaker N, El-Sheikh M, Ramy HA (2012). Pathways to child mental health services among patients in an urban clinical setting in Egypt. Psychiatr Serv.

[41] Marie Bullock B, Lucie Nadeau M, Johanne RM (2012). Spirituality and religion in youth suicide attempters’ trajectories of mental health service utilization: the year before a suicide attempt. J Can Acad Child Adolesc Psychiatry.

[42] Comas-Diaz L (2006). Latino healing: the integration of ethnic psychology into psychotherapy. Psychother Theory Res Pract Train.

[43] Patel V (2011). Invited Commentary: Traditional healers for mental health care in Africa. Glob Health Action.

[44] Uwakwe R, Otakpor A (2014). Public mental health—using the Mental Health Gap Action Program to put all hands to the pumps. Front Public Health.

[45] Lucchetti G, Aguiar PRD, Braghetta CC, Vallada CP, Moreira-Almeida A, Vallada H (2012). Spiritist psychiatric hospitals in Brazil: integration of conventional psychiatric treatment and spiritual complementary therapy. Cult Med Psychiatry.

